# Rhamnose Is Superior to Mannitol as a Monosaccharide in the Dual Sugar Absorption Test: A Prospective Randomized Study in Children With Treatment-Naïve Celiac Disease

**DOI:** 10.3389/fped.2022.874116

**Published:** 2022-04-07

**Authors:** Lori R. Holtz, Julie Hoffmann, Laura Linneman, Mai He, Thomas C. Smyrk, Ta-Chiang Liu, Nurmohammad Shaikh, Cynthia Rodriguez, Roy B. Dyer, Ravinder J. Singh, William A. Faubion

**Affiliations:** ^1^Department of Pediatrics, Washington University School of Medicine, St. Louis, MO, United States; ^2^Department of Pathology & Immunology, Washington University School of Medicine, St. Louis, MO, United States; ^3^Department of Lab Medicine and Pathology, Mayo Clinic, Rochester, MN, United States; ^4^Immunochemical Core Laboratory, Mayo Clinic, Rochester, MN, United States; ^5^Department of Internal Medicine, Mayo Clinic, Rochester, MN, United States

**Keywords:** tissue transglutaminase, dual sugar absorption test, celiac disease, intestinal permeability, Marsh score

## Abstract

**Background and Aim:**

We sought to correlate two different measures of gut permeability [lactulose:mannitol (L:M) and lactulose:rhamnose (L:R)] to the severity of duodenal histopathology in children with and without elevated antibodies to tissue transglutaminase (tTG). A secondary objective was to correlate gut permeability with celiac disease (CD) serology and indices of inflammation and bacterial product translocation.

**Methods:**

We prospectively randomized children undergoing endoscopy with abnormal (*n* = 54) and normal (*n* = 10) concentrations of circulating antibodies to tTG, to either L:M or L:R. Biopsies underwent modified Marsh scoring to measure mucosal injury. Circulating anticore *Escherichia coli* lipopolysaccharide (LPS) IgG, α-1 acid glycoprotein, LPS-binding protein, and C-reactive protein concentrations were measured by enzyme immunoassays.

**Results:**

Of the 54 cases with positive celiac serology, 31 and 69% had modified Marsh 0/1 scores or ≥3a, respectively. Circulating tTG IgA correlated with the modified Marsh score (*p* = 0.03). L:R, but not L:M or percent L excreted, differed according to modified Marsh scores (*p* = 0.01). There was no significant association between any systemic marker of inflammation or gut injury, and modified Marsh scores. Concerningly, most participants had evidence of urinary M before the challenge sugar was administered.

**Conclusions:**

L:R, but not L:M, is associated with modified Marsh scores in children undergoing small bowel biopsy for suspected CD. Despite increased intestinal permeability, we see scant evidence of systemic exposure to gut microbes in these children. Gut permeability testing with L:R may predict which patients with abnormal celiac serology will have biopsy evidence for celiac disease and reduce the proportion of such patients undergoing endoscopy whose Marsh scores are ≤1. M should not be used as a monosaccharide for permeability testing in children.

## Introduction

In a healthy gut, tight junctions maintain a barrier between luminal contents and systemic circulation. The dual sugar absorption test (DSAT) is the classic measure of human intestinal permeability, and uses a disaccharide [lactulose (L)] and a monosaccharide [mannitol (M) or rhamnose (R)] as probes to test the integrity of the barrier. In this test, people are challenged orally with these sugars and urine is assayed to determine the extent to which the sugars are absorbed by the host. However, the choice, concentrations, and volumes of the sugar solutions, timing of the urine collection, and methods of assay vary considerably (1, 2). Furthermore, while M has often been used as the monosaccharide in these assays, its ubiquity as a food and supplement additive is problematic as it is often detected in the urine at baseline [i.e., pre-challenge; (3)]. The ratio of L to R was recently shown to identify increased intestinal permeability in children with environmental enteric dysfunction (2). However, we are unaware of any direct comparison of L and R and L and M in human conditions with increased intestinal permeability.

Celiac disease (CD) is an immune mediated disorder triggered by gluten ingestion in genetically predisposed individuals, which causes villous atrophy and crypt hyperplasia. In North America, diagnosis of CD relies on finding biopsy changes in individuals who have elevated celiac serologic markers. However, discordance between tissue transglutaminase (tTG) levels and biopsy findings are well-known and often lead to diagnostic challenges (4). Because CD patients have increased gut permeability (5), and gut permeability has been recommended as a non-invasive tool to screen for and monitor the response to dietary treatment of CD (5–9), we tested the hypothesis that DSATs using L and R have greater diagnostic precision in CD than L and M.

Here, we compare the performance of the sugars in children undergoing endoscopic upper gastrointestinal mucosal biopsy with elevated and normal concentrations of antibodies to tTG by prospectively randomizing them to receive either L and M, or L and R. We then tested the correlation between L:R, L:M, IgA anti-tTG concentrations, and histologic severity. Given the decreased gut barrier function in CD, we also anticipated that there would be systemic evidence of exposure to gut microbes and inflammation, as has recently been reported (10–15). Therefore, we additionally assayed indices of inflammation and bacterial product translocation to determine if these values correlate with histologic severity, serology, or DSAT signals.

## Methods

### Participants

This study was approved by the Washington University in St. Louis Human Research Protection Office. Informed consent was obtained from parent(s), and assent from children aged 12 years and older. Cases, defined as treatment-naïve (i.e., a gluten-free diet had not yet been started) children ages 0–17.9 years with suspected CD based on an elevated tTG IgA (*n* = 50) or tTG IgG (*n* = 4) who were referred for endoscopy, were eligible to participate. Participants were excluded if they had known inflammatory disorders of the gut or biopsy-proven CD. Controls were recruited from children ages 0–17.9 years with normal celiac serology, and no known inflammatory disorders of the gut. Metadata collected consisted of anthropometrics, laboratory results, medications, past medical history, current symptoms, and last oral intake prior to ingesting the sugars.

### Celiac Serologies

Because tTG IgA serologies lack universal normalized ratios, values were related to the upper limit of normal (ULN) used by the respective laboratories in which each test was performed. These tests were performed at either St. Louis Children's Hospital (66%) or other hospital or commercial laboratories in our region (34%).

### Administration of Dual Sugar Test and Sample Collection

Participants were categorized as cases or controls and then randomized (50:50) to receive either an oral solution consisting of 200 mg of monosaccharide [L-rhamnose (TCI, Portland, OR, USA) or M (Letco, Decatur, AL, USA)] and 1,000 mg of disaccharide [L, Cumberland Pharmaceuticals, Nashville, TN, USA)] in 10 mL of sterile water, prepared by the St. Louis Children's Hospital pharmacy. Fasting participants were provided the solution, and ingestion was monitored and completed within 5 min. Blood was collected when the intravenous line was placed for the anesthesia.

Pre- and 1-h post-dosing urines were collected in a sterile bag or hat depending on the age of the child. If the former was used for collection, a new bag was placed after dosing. If urine was produced <30 min after dosing, an additional bag was placed and this was used for the post-dosing specimen. Start (bag placement) and end (removal) times of bagged specimens and volume of all urines collected were recorded. Urines were kept on ice, volumes determined, and aliquots were frozen (−80°C) until shipped on dry ice to the Mayo Clinic (Rochester, MN) for assay. Sugars were quantified in urine using HPLC MS:MS as previously described (2). These values were used to calculate the concentration of sugars per mL of urine collected, the percent recovery, and L:M and L:R. Percent L was calculated across all participants, and not within groups to which the participants were randomized.

### Biopsies and Interpretation

For each participant with suspected CD two and four biopsies were obtained from the bulb and the second portion of the duodenum, respectively.

All pathologists were blinded to the clinical status of the participants. Two readers initially assigned modified Marsh scores (16) for each biopsy (hereafter described as Marsh scores). If the two scores agreed, we considered this reading to be final. If scores differed by one step (e.g., 3a vs. 3b), the more severe value was used. If scores differed by more than one step (e.g., 3a vs. 3c), a third pathologist was enlisted to read the slide. If two of the three scores agreed, this was used. If all three scores varied, the middle score was used.

### *Escherichia coli* Anticore LPS IgG Enzyme Immunoassay

We used lipopolysaccharide (LPS) from strain F12 (pSK+), a Tn*phoA* mutant of *Escherichia coli* O157:H7 that cannot express the O157 O-side chain (17) to measure seroreactivity to generic (core) *Enterobacteriaceae* LPS. LPS was prepared and characterized, and enzyme immunoassays (EIAs) were optimized and performed, as described (18), with the following changes: each human plasma sample was diluted 1:1,000 in 0.5% BSA in phosphate buffered saline containing 0.05% Tween-20 and added to wells in triplicate. Anti-human IgG F(ab')_2_-horseradish peroxidase (Millipore, Burlington, MA, USA) at a 1:10,000 dilution served as the second antibody. Plates were developed by adding peroxidase substrate tetramethyl benzidine (ThermoFisher, Waltham, MA, USA) (15 min, room temperature). Reactions were stopped with 2M H_2_SO_4_, and absorbance was measured at 450 nm. We constructed a standard curve on each plate using serial dilutions (1:2,000–1:64,000) of serum from a patient recently infected with *E. coli* O157:H7 that was known to be highly reactive to the core LPS. The reactivity of the positive control (1:2,000 dilution) was arbitrarily defined as 1,000 EIA units. All samples were run in triplicate.

### LBP, α-1AGP, and CRP Assays

We used commercial EIAs to measure concentrations of circulating LPS binding protein (LBP) (HyCult, Wayne, PA, USA) and α-1-acid glycoprotein (α-1AGP) and C-reactive protein (CRP) (R&D Systems, Minneapolis, MN, USA), according to the manufacturers' instructions. Values above the upper limit of quantification for CRP were designated as 5,000 ng/mL.

### Statistics

Group differences were analyzed by the Kruskal-Wallis one-way analysis of variance, with *post-hoc* testing and correction for multiple comparisons. Correlation analysis was performed using Spearman's *r*. The significance of differences between continuous and categorical variables were determined by the Mann-Whitney and Fisher's exact test, respectively. Receiver operating characteristic (ROC) curves and area under the curve were calculated to determine the ability of L:M and L:R to distinguish controls from those with Marsh scores of 3a or higher. All *p*-values were two sided, and α-value <0.05 was considered significant. Analyses were performed with Prism 9.3.1 (GraphPad).

## Results

### Demographics

We enrolled 54 cases (50 and four with elevated tTG IgA or tTG IgG, respectively), and 10 controls (Table 1). Endoscopies were performed on controls because of chronic abdominal pain, nausea, and/or vomiting.

**Table 1:** Cohort demographics.

	**Serologic positive participants (n=54)**		**Controls (*n* = 10)**	**All (*n* = 64)**
	**Biopsy positive [*n* = 37 (68.5%)]**	**Biopsy negative [*n* = 17 (31.5%)]**		
Girls (%)	31 (83.8)	9 (52.9)	9 (90)	49 (76.6)
Median age (IQR)—yrs.	9.2 (5.2–12.7)	7.4 (6.5–11.8)	13.0 (11.3–15.7)	9.9 (6.1–12.5)
Race (Caucasian) (%)	36 (97.3)	16 (94.1)	10 (100)	62 (96.9)
Ethnicity (Hispanic)	0	1 (5.9)	0	1 (1.6)
Trisomy 21 (%)	2 (5.4)	1 (5.9)	0	3 (4.7)
Type 1 diabetes (%)	5 (13.5)	4 (23.5)	0	9 (14.1)
IgA deficiency (%)	0	1 (5.9)	0	1 (1.6)
Hypothyroidism (%)	4 (10.8)	1 (5.9)	1 (10)	6 (9.4)
Turner syndrome (%)	1 (2.7)	0	0	1 (1.6)
Randomized to L:M (%)	17 (45.9)	9 (52.9)	6 (60)	32 (50.0)

### Biopsy Results

In one control participant, the normal endoscopy was grossly normal, and the rapid urease test was negative, but *Helicobacter pylori* infection of the stomach was diagnosed on immunostaining.

The Marsh scores assigned by two pathologists agreed perfectly in 27 of the 54 cases, and differed by one step in 19. A third pathologist resolved the scores with greater discrepancies without knowing the values of the first pair of readings. If two of the three scores agreed, that value was used (*n* = 6) (with agreement defined as being identical). If three different values were generated, we used the intermediate score (*n* = 2).

Of the 50 participants with elevated tTG IgA concentrations, 11 (22%) had Marsh 0 and two (4%) had Marsh 1 histology. The remaining Marsh scores were ≥3a. Each of the 4 case participants with abnormal tTG IgG concentrations (but normal tTG IgA) had Marsh 0 (3) or 1 (1) histology. Marsh scores did not vary according to interval between diagnostic serology and biopsies (Kruskal-Wallis *p* = 0.55; Supplementary Table 1). The median (IQR) intervals between obtaining the tTG IgA and biopsy did not significantly differ for children with and without type 1 diabetes [34 (17–62) vs. 42 (40–54) days].

### Serology

**Figure 1:**
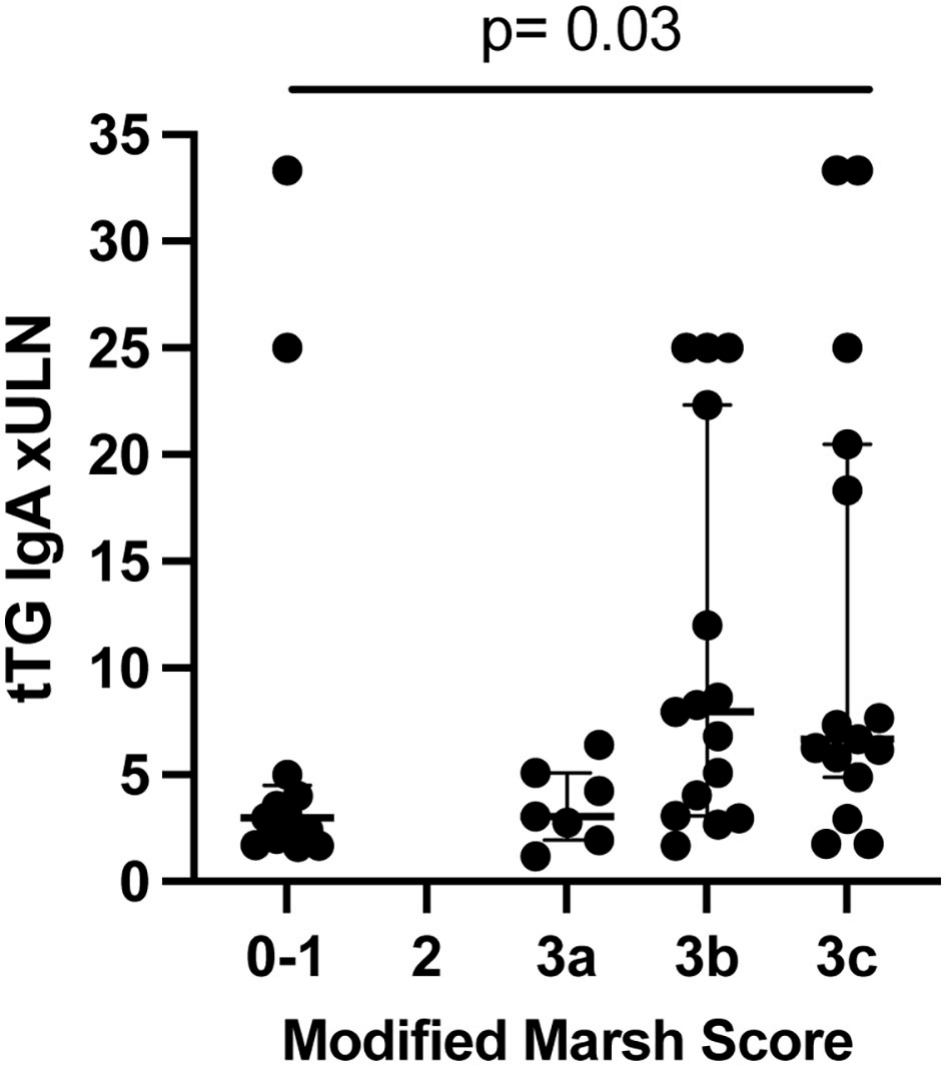
tTG IgA circulating concentration compared to Marsh score in cases (*n* = 50) (not controls). *P*-value from Kruskal-Wallis. Lines show median and quartiles. ULN, upper limit of normal.

Overall, the circulating tTG IgA concentration significantly varied with Marsh scores (Figure 1) among cases (*p* = 0.03). Ancillary celiac screening tests were obtained in a subset of participants at the discretion of the treating physician (Supplementary Table 2). Anti tTG IgG values were normal in four of 14 cases tested. Anti-endomysial antibody was negative in one case participant whose Marsh score was 3b. Anti-deamidated gliadin peptide (DGP) IgA was negative in two of the eight cases in which it was measured. Anti-DGP IgG was elevated in each of the four cases in whom a circulating concentration was determined. The median (IQR) time between type 1 diabetes diagnosis and elevated serology was 2 (1–219) days.

### Sugar Absorption

Six controls and 26 seropositive children were randomized to L and M, while four controls and 28 seropositive cases were randomized to L and R (Supplementary Table 3). One 2 year-old randomized to L:M and one 12 year-old randomized to L:R did not void for over 5 h after ingestion. One 3 year-old randomized to L:R ingested only half of the L:R solution. These three participants were removed from analysis of the sugar probes. Urines were obtained at medians of 65 min (IQR: 60–80) after ingesting sugar in the L:R group, and 67 min (IQR = 60–73) in the L:M group (Mann-Whitney, *p* = ns).

**Figure 2:**
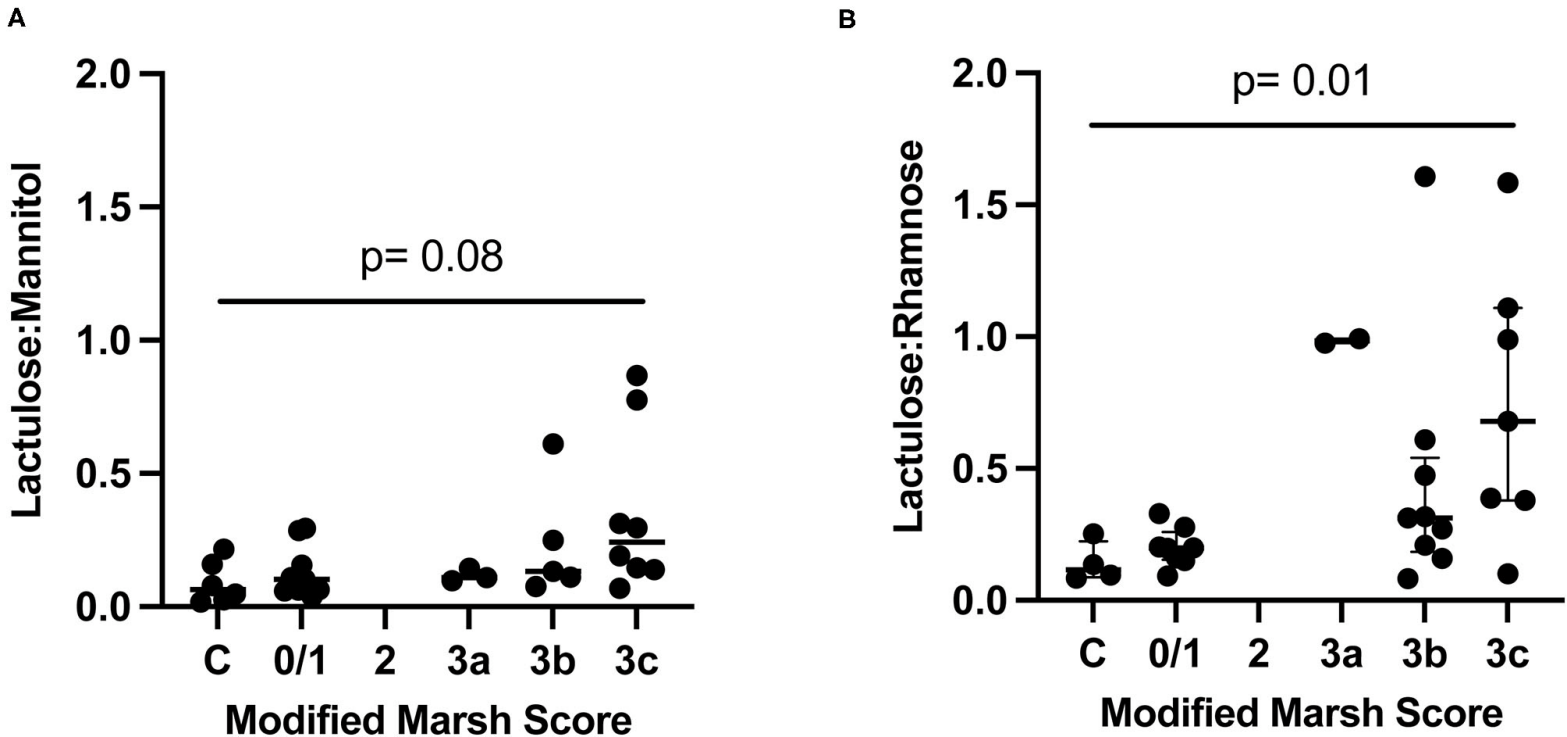
Dual sugar testing compared to Marsh score in controls (C) and cases. *P*-value from Kruskal-Wallis. Lines show median and quartiles. **(A)** Controls and cases randomized to receive L:M. **(B)** Controls and cases randomized to L:R.

L:R (Kruskal-Wallis *p* = 0.01) but not L:M (Kruskal-Wallis *p* = 0.08) significantly differed according to the Marsh scores (Figure 2). We also combined cases with Marsh 2–3C scores and compared those to controls, and again found no significant association with L:M (adjusted *post-hoc* Dunn test *p* = 0.05), but did find an association with L:R (adjusted *post-hoc* Dunn test *p* = 0.01). Additionally, we calculated the area under the curve when comparing controls vs. those with Marsh 3a or higher for both L:M and L:R. The area under the ROC curve (AUC) for L:R was greater than for L:M (0.89 vs. 0.78). We also evaluated the sensitivity and specificity of L:M and L:R at various cutoffs to distinguish controls from those with Marsh scores of 3a or greater. For L:R the following cutoffs give the corresponding sensitivity and sensitivity: 0.098 (sensitivity 94%, specificity 50%), 0.148 (sensitivity 89%, specificity 75%), and 0.262 (sensitivity 78%, specificity 100%). For L:M the following cutoffs give the corresponding sensitivity and sensitivity: 0.059 (sensitivity 100%, specificity 50%), 0.088 (sensitivity 88%, specificity 67%), and 0.232 (sensitivity 38%, specificity 100%). TTG IgA concentrations correlated better with L:R (spearman *r* = 0.409, *p* = 0.03) than with L:M (spearman *r* = 0.382, *p* = 0.04). However, these represent a moderate and weak correlation, respectively.

**Figure 3:**
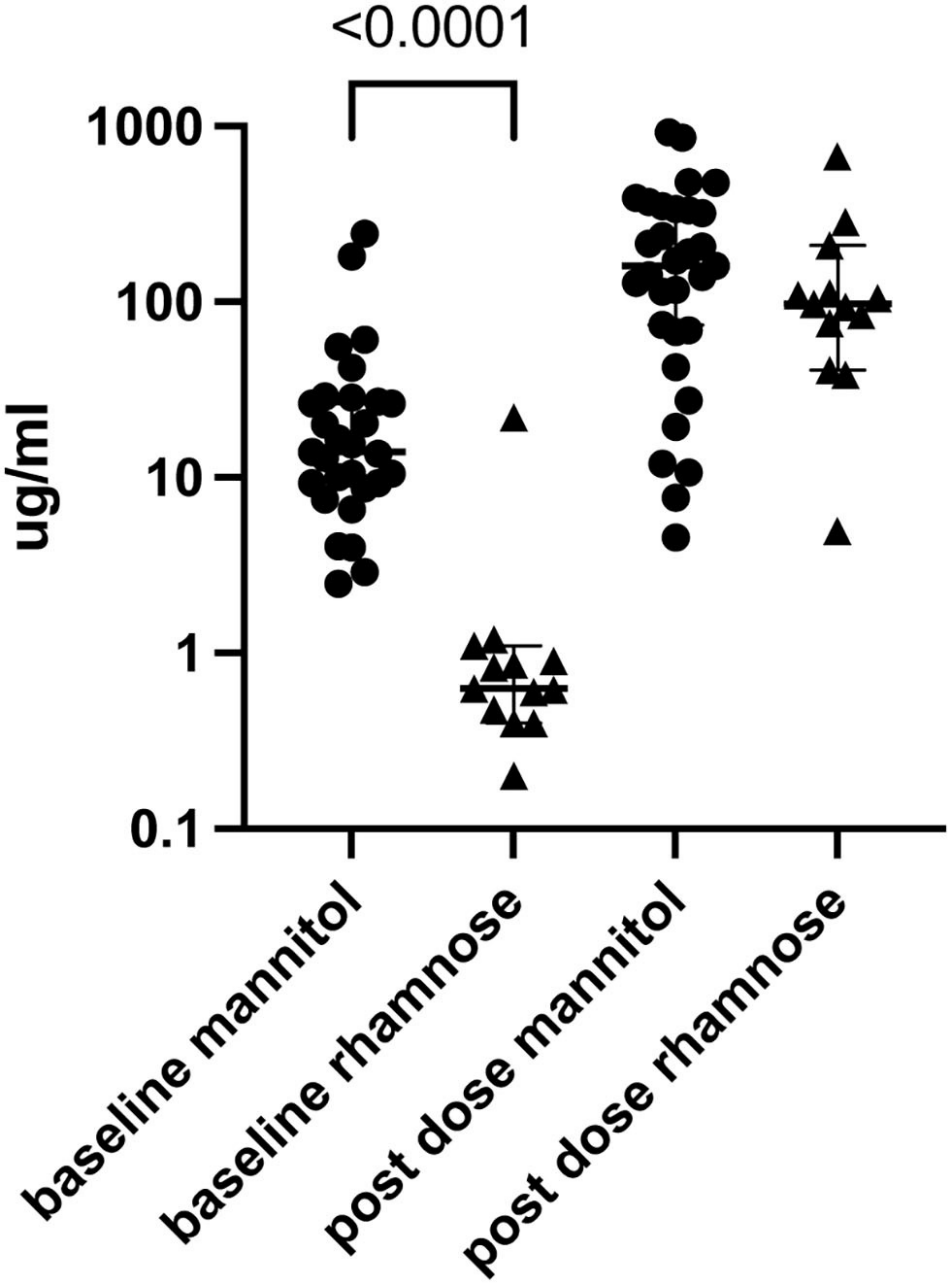
Urine M (*n* = 29) and R (*n* = 13) before and after dual sugar testing. Only participants who produced pre-dose urine and in whom baseline circulating concentrations were detected are shown. Lines show median and quartiles.

We next determined if excretion of single sugars differed according to Marsh score and found no significant association (Supplementary Figure 1). Each of the 29 children tested with L and M who voided before the sugars were administered had detectable M in the urine. Of the 29 children who received L and R and produced pre-dosing urine, 13 (45%) had detectable R (Fisher's exact test *p* < 0.0001). The median (interquartile range) urinary sugar concentrations were considerably greater for M than for the 13 children in whose urines we detected R [14 μg/mL (IQR 9.3–27) vs. 0.63 μg /mL (IQR 0.44–1.0), respectively, *p* < 0.0001; Figure 3].

We asked if subtracting pre-administration urinary M or R improved L:M or L:R association with Marsh scores. Again, there was no significant association with L:M (Kruskal-Wallis *p* = 0.79), but the L:R ratios (Kruskal-Wallis *p* = 0.015) significantly associated (Supplementary Figure 2). We also had the opportunity to estimate the rate of clearance of baseline M by calculating the M in the urine in the post-dosing urine in the children who were tested with L:R, i.e., those who received no M. The median clearance was −6.17 μg/min (IQR −15.3 to −1.6). In two children who received R, the concentration of M actually increased between the pre- and the post-testing urines. This might be attributed to incomplete voiding in the pre-dosing determination, or surreptitious ingestion of a substance containing M soon before the pre-dosing sample was obtained. Using the median M clearance rate, we calculated a corrected cleared M concentration in the children tested with L:M. Again, we found no significant association with this corrected L:M and Marsh score (Kruskal-Wallis *p* = 0.17; Supplementary Figure 3).

### Inflammatory Markers

Blood was obtained at the time of intravenous line placement for upper endoscopy in nine of the 10 controls and 44 of the 54 cases. There were no significant associations between circulating *E. coli* anticore LPS IgG, α-1AGP, LBP, or CRP concentrations and Marsh score or tTG IgA concentration.

## Discussion

We confirmed prior reports that circulating concentrations of anti tTG IgA in the aggregate correlate with Marsh scores (19, 20). However, this correlation was far from absolute. For example, five of the nine participants with circulating anti-tTG IgA values between one and two times the ULN had Marsh scores ≥3a, and seven of the 35 children with circulating anti-tTG IgA concentrations >3 times the ULN had Marsh scores of 0 or 1. One explanation for the discrepancies between Marsh scores of 0/1 and tTG IgA positivity could be self-elimination of gluten between the positive serologic test and the biopsy. However, all participants or their caregivers denied gluten elimination before endoscopy, and the Marsh scores did not vary according to the interval between serology and biopsy. Celiac lesions can be patchy in occurrence (4), so sampling error might have underestimated the degree of mucosal injury. However, the numbers of biopsies obtained from the bulb and second portion of the duodenum was uniform and met current recommendations (4, 21). Furthermore, we cannot be sure that the ULN cut offs in each assay represented the same antibody concentration across sites, because each test kit had its own norm established, and multiples of the ULN might not be equivalent. This is more likely to be a problem near the lower bounds of abnormality, but half of the cases in whom the Marsh score was 0/1 had antibody values to tTG at least triple the upper limit of normal. Finally, we cannot exclude the possibility that the patients with elevated antibodies to tTG and Marsh scores of 0/1 will eventually develop histologic findings typical of CD (22, 23).

It is noteworthy that four out of the nine children with type 1 diabetes had Marsh 0/1 histology. Fluctuations in circulating anti-tTG IgA concentrations without evidence of intestinal changes have been previously described in type 1 diabetes (24). In any event, the mediocre correlation between Marsh score and antibody concentrations to tTG at the critical first biopsy highlights the need for additional methods to predict CD and inform decisions to perform or forego an endoscopic biopsy.

We sampled urine 1 h after ingestion, a protocol used in children with environmental enteric dysfunction (2) and children with diagnosed CD (25), to avoid truncated collection times, but it is possible that we did not capture the peak of the filtered sugar columns (26).

Previous studies have raised concern about the presence of pre-testing (baseline) M (2, 3) in the urine, most likely from dietary sources. Indeed, pre-dosing urinary M was universal in our cohort. In contrast, baseline R urinary concentrations, when detected, were substantially less than baseline M concentrations, and, when present, were considerably less than the concentrations of the monosaccharides in the post-dosing urines (Figure 3). Furthermore, we were unable to improve the performance of the L:M test by subtracting the baseline M or adjusting the M concentration according to the M clearance determined in the R group. Based on our data, L:R better predicts histopathology in treatment-naïve CD, and because of the pre-dosing presence of M in these children, we cannot endorse using M in the DSAT.

Our inability to find evidence of systemic inflammation in treatment-naïve CD is somewhat surprising, in view of the associations between CD and extra-intestinal disorders, including hepatopathy, arthritis, thyroiditis, and dermatitis. A recent study from Buenos Aires found that CRP values were significantly higher in adult patients with newly diagnosed CD than in controls (15), though most association occurred within a range that would be considered normal. In addition to age, other potential differences between our cohort and the Argentinean cohort include duration of symptoms and degree of Marsh scores, but these data were not provided in the adult cohort. Moreover, by definition, adults diagnosed with CD will have had longer pre-biopsy exposure to gluten. Also unexpectedly, despite increased intestinal permeability, the children in this study with diet naïve CD lacked evidence of systemic exposure to LPS. Perhaps this is related to the lower microbial burden in the proximal small bowel where CD is believed to be most active.

In conclusion, in the first comparison of L:R and L:M to identify correlates to disease severity, we demonstrate superiority of L:R. Our data recommend that L:M testing not be performed. Further work is needed to improve our ability to predict which patients with abnormal celiac serology will have biopsy evidence of active CD, but based on the L:R scores, assessment of intestinal permeability might reduce unnecessary endoscopies. Additionally, it will be useful to determine if L:R correlates with tissue healing after a gluten free diet is initiated, in which case permeability testing might supplant celiac serology, which can be problematic as a measure of disease monitoring (27).

## Data Availability Statement

The original contributions presented in the study are included in the article/Supplementary Material, further inquiries can be directed to the corresponding author/s.

## Ethics Statement

The studies involving human participants were reviewed and approved by Washington University in St. Louis Human Research Protection Office. Written informed consent to participate in this study was provided by the participants' legal guardian and assent from children aged 12 years and older.

## Author Contributions

LH and WF conceived and designed the experiments, analyzed the data, and wrote the manuscript. JH and LL recruited the participants and collected the clinical data and samples. MH, TS, and T-CL scored the biopsies. CR, NS, RD, and RS generated and interpreted data. All authors contributed to the article and approved the submitted version.

## Funding

This work was supported in part by the Bill & Melinda Gates Foundation (OPP1066153), the Children's Discovery Institute (MD-FR-2013-292), and P30DK052574 (for the Biobank Core). Study sponsors had no role in study design, collection, analysis, and interpretation of data, writing of this report, or the decision to submit for publication.

## Conflict of Interest

WF consults for MediBeacon without personal compensation. T-CL consults for Interline Therapeutics and holds a research grant from Pfizer. The remaining authors declare that the research was conducted in the absence of any commercial or financial relationships that could be construed as a potential conflict of interest.

## Publisher's Note

All claims expressed in this article are solely those of the authors and do not necessarily represent those of their affiliated organizations, or those of the publisher, the editors and the reviewers. Any product that may be evaluated in this article, or claim that may be made by its manufacturer, is not guaranteed or endorsed by the publisher.

## Supplementary Material

Supplementary Figure 1 Percent recovery in urine of **(A)** L in children challenged with L and M and L and R, **(B)** M in children challenged with L and M, and **(C)** R in children challenged with L and R. Horizontal lines represent medians and quartiles. Kruskal-Wallis is not significant for all comparisons.

Supplementary Figure 2 Dual sugar testing (baseline subtracted) compared to Marsh score in controls (C) and cases. *P*-values from Kruskal-Wallis. Horizontal lines represent medians and quartiles. **(A)** Controls and cases randomized to receive L:M. **(B)** Controls and cases randomized to L:R.

Supplementary Figure 3 Corrected L:M (adjusted M concentration based on the M clearance determined in the R group) compared to Marsh score controls (C) and cases. Horizontal lines represent medians and quartiles. *P*-values from Kruskal-Wallis.

Supplementary Table 1 TTG IgA and biopsy results in cases.

Supplementary Table 2 Additional serological testing in cases.

Supplementary Table 3 Characteristics of cases and controls.

## Abbreviations

α-1AGP: α-1-acid glycoprotein
CRP: C-reactive protein
CD: celiac disease
DSAT: dual sugar absorption test
EIAs: enzyme immunoassays
L: lactulose
LPS: lipopolysaccharide
LBP: LPS binding protein
M: mannitol
R: rhamnose
tTG: tissue transglutaminase
ULN: upper limit of normal.
