# Supplementary figures and images for: Rhamnose Is Superior to Mannitol as a Monosaccharide in the Dual Sugar Absorption Test: A Prospective Randomized Study in Children With Treatment-Naïve Celiac Disease
Source: Front Pediatr. 2022 Apr 7;10:874116. doi: 10.3389/fped.2022.874116 (PMC9021878; doi:10.3389/fped.2022.874116)

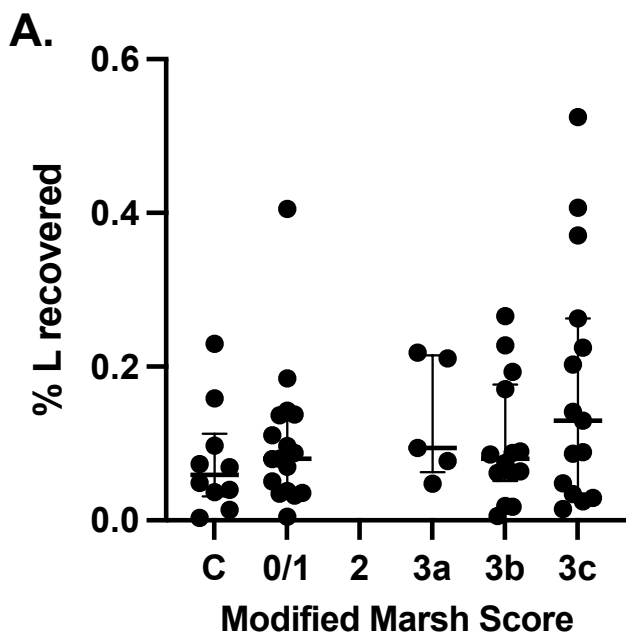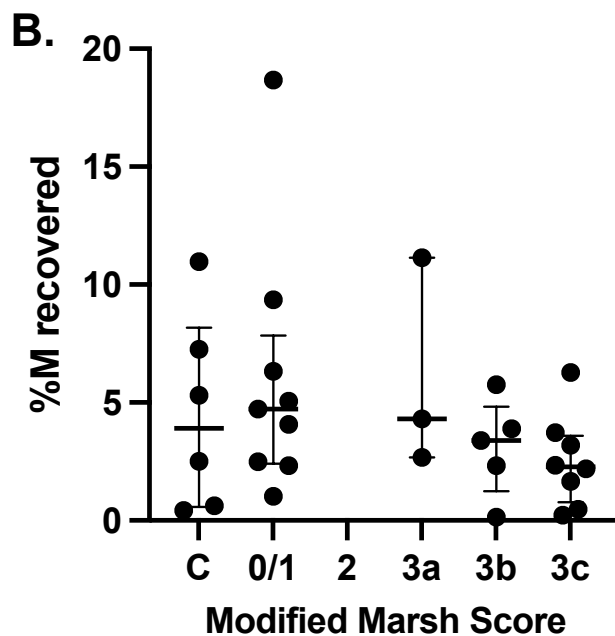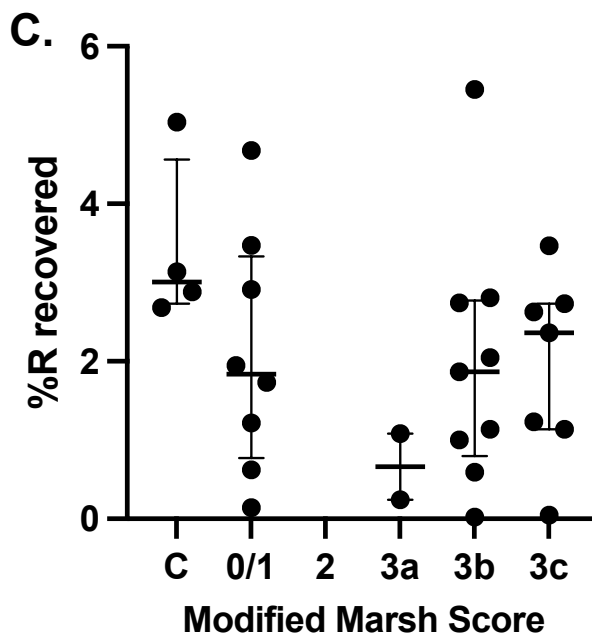

Supplement: Supplementary Figure 1 — Percent recovery in urine of (A) L in children challenged with L and M and L and R, (B) M in children challenged with L and M, and (C) R in children challenged with L and R. Horizontal lines represent medians and quartiles. Kruskal-Wallis is not significant for all comparisons. [file Data_Sheet_1.PDF]

**A.**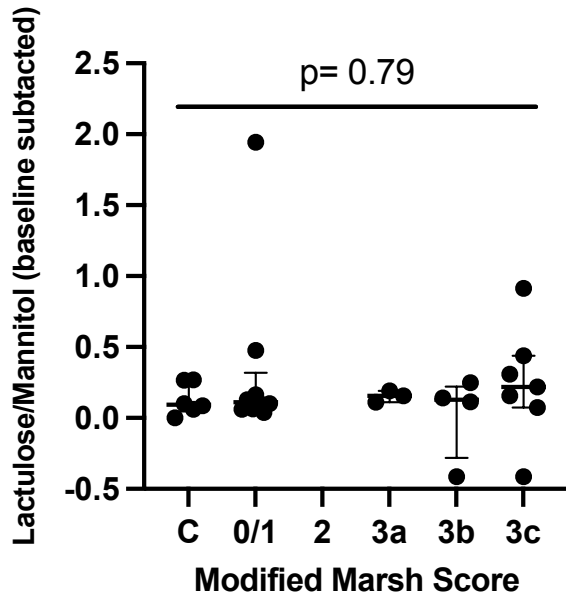**B.**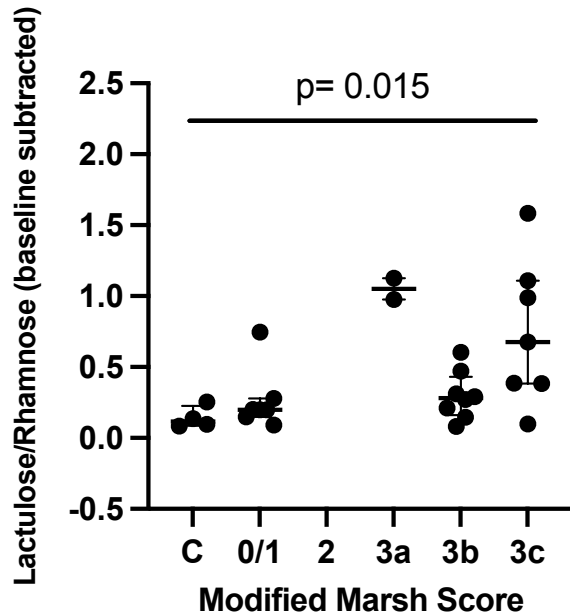

Supplement: Supplementary Figure 2 — Dual sugar testing (baseline subtracted) compared to Marsh score in controls (C) and cases. P-values from Kruskal-Wallis. Horizontal lines represent medians and quartiles. (A) Controls and cases randomized to receive L:M. (B) Controls and cases randomized to L:R. [file Data_Sheet_2.PDF]

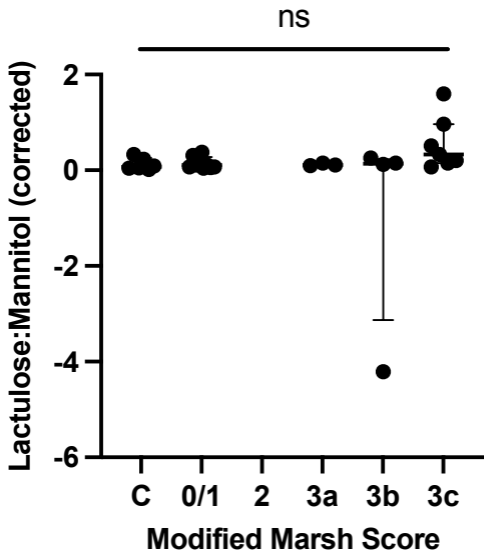

Supplement: Supplementary Figure 3 — Corrected L:M (adjusted M concentration based on the M clearance determined in the R group) compared to Marsh score controls (C) and cases. Horizontal lines represent medians and quartiles. P-values from Kruskal-Wallis. [file Data_Sheet_3.PDF]
