# Supplementary material for: Rhamnose Is Superior to Mannitol as a Monosaccharide in the Dual Sugar Absorption Test: A Prospective Randomized Study in Children With Treatment-Naïve Celiac Disease
Source: Front Pediatr. 2022 Apr 7;10:874116. doi: 10.3389/fped.2022.874116 (PMC9021878; doi:10.3389/fped.2022.874116)
Supplement: Supplementary Table 1 — TTG IgA and biopsy results in cases. [file Table_1.DOCX]

| Supplemental Table 1. TTG IgA and biopsy results in cases | | | |
| --- | --- | --- | --- |
| Variable (median (IQR)) or no. (percent) | 1-30 days between initial anti-tTG IgA and biopsy n=21 | 31-60 days between initial anti-tTG IgA and biopsy n=18 | >61 days between initial anti-tTG IgA and biopsy n=11 |
| Days between initial anti-TTG IgA and biopsy | 17 (12-24) | 41 (37-46) | 73 (69-87) |
| Initial anti-TTG IgA (x ULN) | 5.1 (3.0-8.0) | 4.5 (3.0-7.6) | 6.67 (2.4-25) |
| Marsh scores  0/1  2  3 | 4 (19)  0 (0)  17 (81) | 6 (33)  0 (0)  12 (67) | 3 (27)  0  8 (73) |
