# Supplementary material for: Rhamnose Is Superior to Mannitol as a Monosaccharide in the Dual Sugar Absorption Test: A Prospective Randomized Study in Children With Treatment-Naïve Celiac Disease
Source: Front Pediatr. 2022 Apr 7;10:874116. doi: 10.3389/fped.2022.874116 (PMC9021878; doi:10.3389/fped.2022.874116)
Supplement: Supplementary Table 2 — Additional serological testing in cases. [file Table_2.DOCX]

| Supplemental Table 2. Additional Serological Testing in Cases | | | | |
| --- | --- | --- | --- | --- |
| Modified Marsh Score (n) | tTG IgG  positive/tested (percent) | Anti-endomysial IgA positive/tested (percent) | Anti-deamidated gliadin IgA positive/tested (percent) | Anti-deamidated gliadin IgG  positive/tested (percent) |
| 0/1 (17) | 5/7 (71) | 2/2 (100) | 1/1 (100) | 0/0 |
| 3A (7) | 1/1 (100) | 2/2 (100) | 1/1 (100) | 1/1 (100) |
| 3B (15) | 2/3 (67) | 4/5 (80) | 1/3 (33) | 1/1 (!00) |
| 3C (15) | 2/3 (67) | 8/8 (100) | 3/3 (100) | 2/2 (100) |
