# Supplementary material for: Rhamnose Is Superior to Mannitol as a Monosaccharide in the Dual Sugar Absorption Test: A Prospective Randomized Study in Children With Treatment-Naïve Celiac Disease
Source: Front Pediatr. 2022 Apr 7;10:874116. doi: 10.3389/fped.2022.874116 (PMC9021878; doi:10.3389/fped.2022.874116)
Supplement: Supplementary Table 3 — Characteristics of cases and controls. [file Table_3.DOCX]

| Supplemental Table 3. Characteristics of cases and controls | | | | |
| --- | --- | --- | --- | --- |
| Variable (median IQR or no. (percent) | Cases randomized to receive L:M (n=26^2^) | Cases randomized to receive L:R (n=28^3^) | Controls randomized to receive L:M (n=6) | Controls randomized to receive L:R (n=4^4^) |
| Age | 7.6 (5.2-12.2) | 9.1 (6.1-11.8) | 15.3 (12.9-16.7) | 11.7 (8.9-12.8) |
| Girl | 20 (77.0) | 20 (71.4) | 5 (83.3) | 4 (100) |
| Race (Caucasian) | 26 (100) | 26 (93.0) | 6 (100) | 4 (100) |
| Ethnicity (Hispanic) | 0 | 1 (3.6) | 0 | 0 |
| Baseline monosaccharide detected (no. positive/no.tested) | 23/23 (100) | 11/25 (44) | 6/6 (100) | 2/4 (50) |
| Baseline monosaccharide concentrations among those with monosaccharide detected (µg/mL) | 12.9 (9.0-20.2) | 0.83 (0.44-1) | 26.8 (26.4-28.5) | 0.61 (0.60-0.61) |
| Difference in mannitol concentrations (pre-dosing urine – post-dosing urine) after receiving L:R (µg) | NA | -722.1 (-1363.2 to -183.4)  n=15 | NA | 26.9 (-29.0-82.7)  n=2 |
| Interval between pre-dosing urine and post-dosing urine^1^ (min) | NA | 66 (60-81) | NA | 63 (62-64) |
| Marsh scores  0/1  2  >3 | 9 (34.6%)  0 (0%)  17 (65.4%) | 8 (28.6%)  0 (0%)  20 (71.4%) | 6 (100%)  0 (0%)  0 (0%) | 3 (75%)  0 (0%)  1 (25%) |
| Anti-tTG IgA (x ULN) | 4.5 (2.9-18.9)  n=24 | 5.5 (2.8-8.2)  n=26 | 0.18 (0.15-0.21)  n=6 | 0.19 (0.17-0.21)  n=4 |
| Interval between anti-tTG IgA determination and dual sugar absorption testing (days) | 36.5 (20-60.5) | 37.5 (18.8-53.5) | 39 (22.5-46.5) | 39 (35-46.8) |
| Anti-*E. coli* core LPS (units) | 42.2 (39.3 -82.7) | 49.3 (37.7-96.5) | 41.3 (41.1-43.7) | 58.2 (39.6-81.6) |
| LBP (µg/mL) | 10.5 (7.8-19.3) | 15.6 (10.5-17.8) | 18.1 (12.7-19.8) | 15.6 (12.7-17.7) |
| α-1-AGP (µg/mL) | 626 (510-873) | 572 (480-629) | 741 (727-784) | 558 (487-629) |
| CRP (ng/mL) | 272.7 (138.9-600.0) | 525.9 (215.4-1000) | 334.4 (97.1-549.7) | 268.7 (223.9-275.1) |
| L:R | NA | 0.32 (0.20-0.98) | NA | 0.12 (0.09-0.17) |
| L:M | 0.14 (0.10-0.29) | NA | 0.06 (0.03-0.14) | NA |
| Percent L excreted | 0.09 (0.05-0.14) | 0.09 (0.04-0.21) | 0.04 (0.02-0.06) | 0.09 (0.07-0.11) |
| Percent R excreted | NA | 1.8 (0.9-2.8) | NA | 3.0 (2.8-3.6) |
| Percent M excreted | 3.4 (2.3-5.1) | NA | 3.9 (1.1-6.8) | NA |
| Post-dosing monosaccharide in urine (µg/mL) | 192 (73.6-351) | 83.0 (35.6-132.8) | 93 (49.0-135.8) | 139.5 (90.1-308) |
| Post-dosing disaccharide in urine (µg/mL) | 20 (9.8-38) | 23.5 (9.9-44.3) | 4.6 (3.4-9.6) | 18.6 (14.1-38.9) |
| ^1^This value relates to the clearance of the baseline mannitol in children who received rhamnose as the monosaccharide.  ^2^ 1 child did not void in this category, so their sugar excretion values cannot be calculated. Other variables are, however, entered into analysis. Blood available for 21 children.  ^3^ 1 child did not void and 1 child only drank half of the sugar solution in this category, so their sugar excretion values cannot be calculated. Other variables are, however, entered into analysis. Blood available for 23 children.  ^4^Blood available for 3 children | | | | |
